# The MinCDJ System in *Bacillus subtilis* Prevents Minicell Formation by Promoting Divisome Disassembly

**DOI:** 10.1371/journal.pone.0009850

**Published:** 2010-03-24

**Authors:** Suey van Baarle, Marc Bramkamp

**Affiliations:** Institute for Biochemistry, University of Cologne, Cologne, Germany; BMSI-A*STAR, Singapore

## Abstract

**Background:**

Cell division in *Bacillus subtilis* takes place precisely at midcell, through the action of Noc, which prevents division from occurring over the nucleoids, and the Min system, which prevents cell division from taking place at the poles. Originally it was thought that the Min system acts directly on FtsZ, preventing the formation of a Z-ring and, therefore, the formation of a complete cytokinetic ring at the poles. Recently, a new component of the *B. subtilis* Min system was identified, MinJ, which acts as a bridge between DivIVA and MinCD.

**Methodology/Principal Findings:**

We used fluorescence microscopy and molecular genetics to examine the molecular role of MinJ. We found that in the absence of a functional Min system, FtsA, FtsL and PBP-2B remain associated with completed division sites. Evidence is provided that MinCDJ are responsible for the failure of these proteins to localize properly, indicating that MinCDJ can act on membrane integral components of the divisome.

**Conclusions/Significance:**

Taken together, we postulate that the main function of the Min system is to prevent minicell formation adjacent to recently completed division sites by promoting the disassembly of the cytokinetic ring, thereby ensuring that cell division occurs only once per cell cycle. Thus, the role of the Min system in rod-shaped bacteria seems not to be restricted to an inhibitory function on FtsZ polymerization, but can act on different levels of the divisome.

## Introduction

Cell division in rod-shaped bacteria generates two equally sized daughter cells and thus requires the formation of a septum precisely at midcell. This process is carried out by a highly complex protein machinery called the divisome, which is currently thought to encompass approximately 18 proteins of which many are conserved among different bacteria [1], [2], [3], [4]. Cell division begins with the formation of the Z-ring, which subsequently recruits a number of proteins. The fully assembled divisome then initiates synthesis of a new cell wall and invagination of the cell membrane. After septation is complete, the divisome is disassembled.

The Z-ring, around which bacterial division is centered, is composed of the bacterial tubulin homologue, FtsZ [4], [5]. In the presence of GTP, FtsZ polymerizes into protofilaments which, through lateral interactions, can assemble into a ring-like structure [6], [7], [8]. A number of proteins promote assembly of FtsZ into the Z-ring, including FtsA, ZapA, and SepF (which is exclusively found in Gram-positive bacteria) [4], [9], [10], [11]. FtsA is an actin homologue with a twofold function, namely to promote FtsZ polymerization by bringing FtsZ polymers to the membrane, and to recruit late divisome proteins to the Z-ring [12], [13], [14]. Both ZapA and SepF promote assembly of the Z-ring, but are not essential for septum formation [9], [10], [15]. Later recruits to the divisome are all membrane-spanning proteins, most of which have a major extracellular domain [4]. However, a specific biochemical function has only been assigned to PBP-2B (FtsI in *E. coli*), which catalyzes the transpeptidation reaction during synthesis of new peptidoglycan for the growing cell wall [16].

Cell division is subject to both spatial and temporal regulation. In rod-shaped bacteria, the Min system and nucleoid occlusion both ensure that division takes place precisely at midcell. Nucleoid occlusion prevents septum formation over the nucleoid through the action of the DNA-binding proteins Noc (in *B. subtilis*) and SlmA (*E. coli*) [17], [18]. Meanwhile, the Min system inhibits Z-ring formation at the cell poles [19]. This system has been well described for *E. coli*, where it consists of three proteins: MinC, MinD and MinE [20], [21]. The actual inhibitor of Z-ring formation is MinC, which functions as a dimer and consists of two functional domains: an N terminal domain, which is implicated in FtsZ interaction, and a C-terminal domain that interacts with MinD [22]. Although MinC has been shown to inhibit FtsZ polymerization directly, there are also a number of reports which suggest that MinC actually prevents lateral interactions between filaments, thereby inhibiting Z-ring formation [8], [23], [24]. MinD is a membrane-associated ATPase that sequesters MinC to the membrane interface, allowing it to interact with FtsZ [25]. The third protein, MinE, imparts topological specificity by stimulating MinCD oscillation, thereby ensuring that the concentration of MinCD is highest at the poles [26]. MinE does this by binding to the trailing edge of MinD [27] and stimulating its ATP hydrolysis, which results in the release of MinD, and thus MinC and MinE, from the membrane [26]. The redistribution of MinD seems to follow a spiral like pattern [28], which may have a lipid dependency [29]. *B. subtilis* contains homologues of MinCD, but not MinE. Instead, DivIVA acts as the topological factor in this system, being constantly associated with the cell poles, and was believed to target MinCD [30], [31], [32], [33]. In this model, and in contrast to *E. coli*, the system is assumed to be mainly static, with the majority of MinC and MinD remaining at the poles. The combined action of nucleoid occlusion and the Min system ensures that cytokinesis only occurs at midcell, after segregation of the nucleoids, and therefore also contributes to temporal regulation of cell division [34].

The traditional model of Min system function has recently been challenged by a number of discoveries. First of all, it was shown that the static localization pattern observed in *B. subtilis* was caused by overexpression of MinC and that MinC-GFP localization is much more dynamic, localizing to the division site before visible constriction [35]. It also appears that the poles are actually a secondary localization site for MinC, with the protein mostly localized to active division sites. Interestingly, it was also shown that in the absence of MinCD, the timing of cell division is defective [35]. Additionally, a fourth component of the Min system was discovered, MinJ, which acts as a bridge between DivIVA and MinD, and is thus the actual sequestrator of MinCD to the poles [36], [37]. DivIVA was shown to be necessary for MinJ localization. Strikingly, in the absence of MinJ, FtsZ-GFP structures are visible between segregated nucleoids [36]. However, GFP-PBP-2B and GFP-FtsL fail to localize in the absence of MinJ. This indicates that the Min system is not only involved in inhibiting aberrant division at the poles, but may also play a role in the assembly/disassembly of a functional divisome.

In this paper we show that MinJ preferentially localizes to sites of division instead of being present at the poles and the site of division at the same time. In the absence of MinC, MinD or MinJ, components of the cytokinetic ring, including FtsA, FtsL and PBP-2B, remain associated with the young poles. Based on localization studies and protein stability studies with PBP-2B we conclude that in *min* mutants the divisomes fail to disassemble properly after completion of septation. Overexpression of MinD in the absence of MinJ results in lethal filamentation, indicating that the Min system is able to inhibit formation of a complete cytokinetic ring by preventing the membrane components to associate with the Z-ring. The failure of the cytokinetic ring to disassemble allows it to initiate a new round of replication, leading to minicell formation. Our results provide strong evidence for a new mode of action with which the Min system prevents minicell formation downstream of FtsZ assembly, ensuring that division occurs only once in every cell cycle.

## Results

### MinJ localizes preferentially to late septa

**Figure 1:**
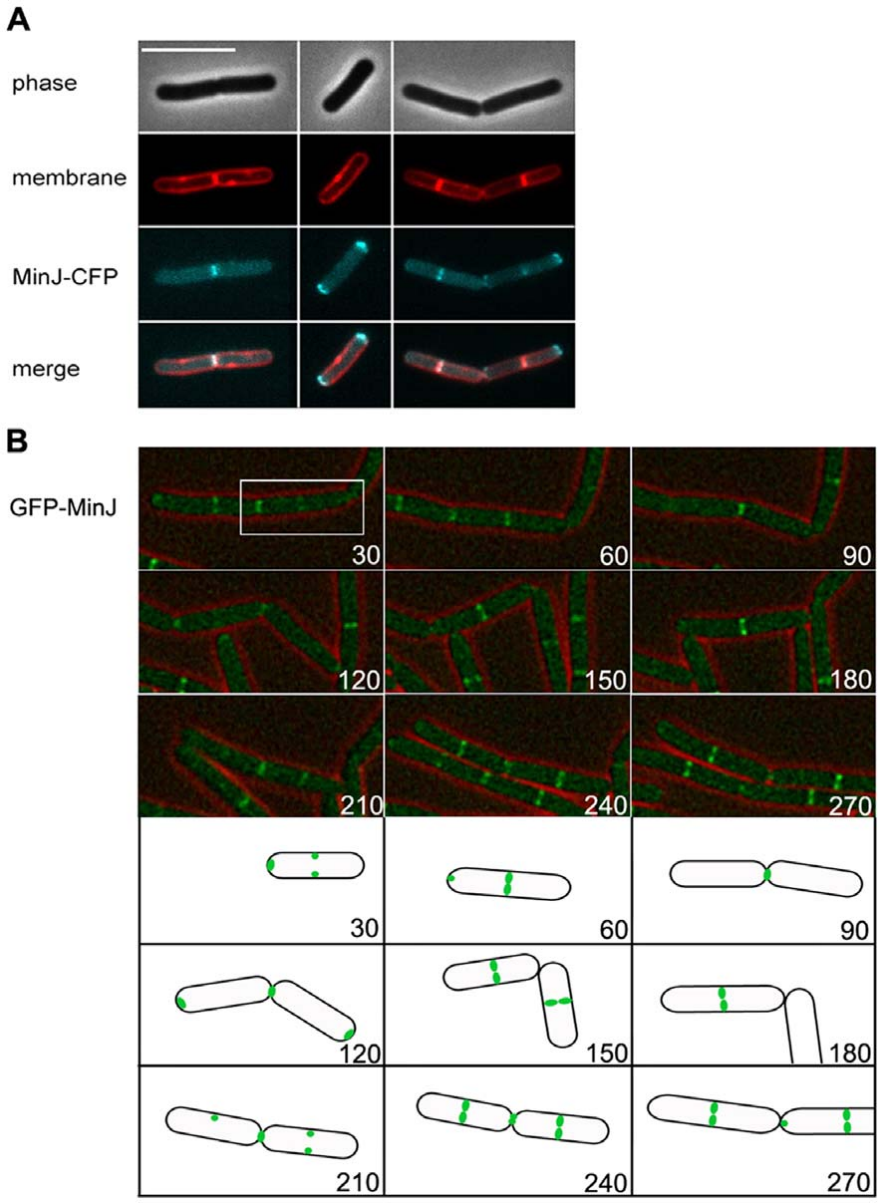
Dynamic localization of MinJ. **A**. Localization of MinJ-CFP, expressed from its native locus (strain SB003). From top to bottom the images show the phase contrast, membrane stain (FM4-64), MinJ-CFP, and merged image of membrane and MinJ-CFP. The scale bar is 5 µm. Three different localization patterns are shown: on the left panel, MinJ-CFP localizes at young poles/midcell (44.6%), in the middle panel, MinJ-CFP localizes to both poles (37.6%), and in the right panel, MinJ-CFP localizes to both midcell and poles (17.8%). In total 250 cells were counted. **B**. Time lapse microscopy of GFP-MinJ (strain MB001) showing the dynamic localization of MinJ. Numbers indicate minutes. Top shows a merged image of phase contrast and GFP-MinJ microscopy image, the bottom part shows a cartoon of localization of GFP-MinJ of one particular cell (highlighted in white box in microscopy image). The image shows that the localization of GFP-MinJ depends on the state of the cell cycle. When cells are not dividing, GFP-MinJ is localized to the poles. As cells prepare to divide, GFP-MinJ moves from the poles to midcell. After division is completed, GFP-MinJ moves back to the poles. The complete movie can be seen in the supplemental material (Movie S2).

The subcellular localization of MinJ has been described before [36], [37]. However, in our previous study we used an inducible copy of GFP-MinJ. In order to avoid false localization due to overexpression, we constructed a strain expressing MinJ-CFP from its native promoter by using plasmid pSG1186 [38], which resulted in strain SB003 (a similar approach was used by Patrick and Kearns, 2008). SB003 cells had a normal cell length and produced a minimal amount of minicells (≤3%), indicating that the protein is at least partial functional. Cells growing at exponential phase were analyzed microscopically. Three distinct patterns of localization could be observed. For the sake of clarity, recently completed division sites are denoted as new poles (a membrane stain was used to distinguish ongoing and completed septation), while other poles are referred to as old poles. We observed that MinJ-CFP was mostly localized at a pole, whether old or new. 44.6% of the cells showed a band of MinJ-CFP at a late division site or a new pole (Fig. 1, left panel), with no MinJ-CFP seen at the old poles. 37.6% of cells showed MinJ-CFP localized only at the old poles (Fig. 1, middle panel). And lastly, only 17.6% of cells showed MinJ-CFP localization at both the new and old poles (Fig. 1, right panel). With the inducible copy of MinJ-GFP, we found that 26% of cells had a MinJ-GFP band only at a late division site/new pole, 33% of cells showed MinJ-GFP at only the old poles, and 42% of the cells showed MinJ-GFP at both old poles as well as new poles. Thus, MinJ-CFP clearly prefers to localize either to only the old poles or the new, but not both. The results obtained with MinJ- CFP is similar to that of MinC4-GFP expressed from its native promoter, which localizes preferentially to midcell but is recruited to the cell poles during intermediate stages of FtsZ depletion [35], [36], [37]. Thus, MinJ either binds to the cell poles or the site of septation, but seldom to both sites simultaneously.

We then used time lapse microscopy to determine the dynamics of MinJ. For this, we used strain MB001, where GFP-MinJ is expressed from the *amyE* locus under control of *P_xyl_*. A knock-in MinJ-CFP strain was not used as the fluorescence signal was not sufficient for time-lapse microscopy. Strain MB001 was induced with only 0.1% xylose to achieve a low level of induction, which gave the similar distribution patterns as we observed with the MinJ-CFP variant described above. Figure 1B shows that GFP-MinJ is present at the old poles and, as cell division occurs, it moves from the old poles to midcell, indicating that the localization sites are not oversaturated (Figure 1B, 60, 150 minutes). Following cell division, GFP-MinJ stays associated with what has become a new pole, but also moves back to the old poles (Fig. 1B, 120 and 210 minutes). The localization of MinJ depends on the state of the cell cycle. When no cell division is occurring, MinJ is localized to both poles. When cell division is in its late stages, MinJ moves from the poles to the septum. After cell division, MinJ is localized at the septum, corresponding to the new pole. Recently, it was postulated that the main site of minicell formation, and thus the most important site of preventing Z-ring formation, is at the new pole [35]. This implies that it is imperative for the components of the Min system to move to sites of cell division in order to protect these new poles. The localization pattern of MinJ-CFP supports this postulation.

### FtsA-YFP remains associated with the poles in cells Min-deficient cells

Previously, it was shown that in the absence of MinJ, FtsZ-rings do develop at regular intervals [36] (and see Movie S1). In order to get more insight into the action of different components of the Min system on the formation and maturation of Z-rings, we introduced an IPTG-inducible copy of FtsA fused to YFP (FtsA-YFP) in wild type cells (strain SB067), Δ*minCD* (strain SB060) and Δ*minJ* (SB066). FtsA-YFP was used for this purpose as a marker for Z-rings, as this protein always associates with FtsZ and FtsA-YFP is functional while a GFP tagged FtsZ is not. Expression of FtsA-YFP was always kept to a minimum and started only 2 h before the cells were examined. It should also be emphasized that this expression procedure in wild type background did not lead to division phenotypes. Thus, only conditions under which expression of FtsA-YFP had no influence on division and viability were used.

**Figure 2:**
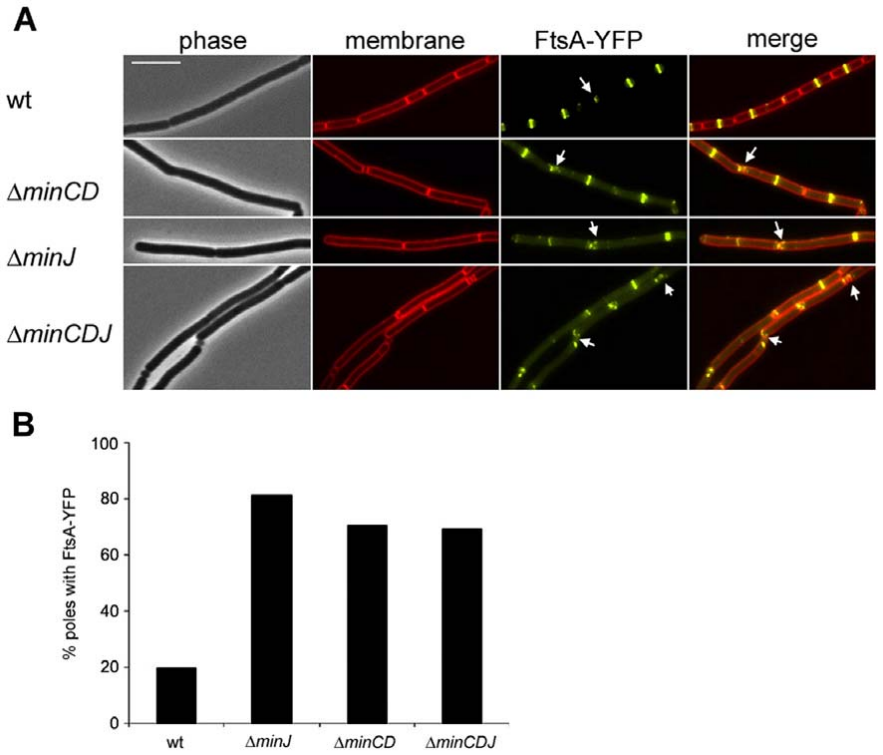
FtsA-YFP remains associated with the cell poles in the absence of a functional Min system. **A**. From top to bottom, localization of FtsA-YFP in wild type cells (SB067), Δ*minCD* (SB060), Δ*minJ* (SB066), and Δ*minCDJ* (SB061). Left to right: phase contrast image, membrane stain (FM4-64), FtsA-YFP, and a merged image of the membrane stain and FtsA-YFP. Scale bar is 5 µm. Arrows indicate FtsA rings resembling spirals, which are found at cell poles/late septa. **B**. Percentage of cell poles containing FtsA-YFP in wild type cells (SB067), Δ*minCD* (SB060), Δ*minJ* (SB066), and Δ*minCDJ* (SB061) (n = 200).

In wild type cells, FtsA-YFP forms compact rings, localized precisely at midcell. On rare occasions FtsA can be seen at late septa (Fig. 2A, wt, arrow). However, in the absence of MinJ, FtsA-YFP rings are more often polar localized (Fig. 2A, Δ*minJ*). Many of the polar FtsA structures appear to be short helices, as was previously described for FtsZ in the Δ*minJ* strain [36]. These short helices can also be seen in the absence of MinCD. These helix structures appear most frequently at the poles (2A, Δ*minCD* and Δ*minJ* arrows). Most interestingly was the frequency with which FtsA-YFP rings appeared. In wild type cells FtsA-YFP is exceedingly regularly distributed at midcell. In Δ*minJ*, FtsA-YFP rings appear to be as frequent as in wild type, although quite often two rings can be visualized very close to recently completed septa, suggesting that spatial control of ring formation is deficient in these strains. Due to both polar localized FtsA-YFP rings and the filamentous phenotype of Δ*minJ* cells, it was quite common to see a single cell with multiple FtsA-YFP rings. Δ*minCD* cells are slightly filamentous, although not to the same extent as a Δ*minJ* strain, consequently in this strain numerous cells could be visualized, which contained multiple FtsA-YFP rings. In contrast to wild type, where FtsA-YFP is mostly found at midcell, we observed that in Δ*minCD* and Δ*minJ*, FtsA-YFP was very frequently found at the newly formed poles. This polar localization of FtsA structures could either be due to a reduced disassembly of divisomes, or to an immediate reassembly of divisomes close to the cell poles. We therefore determined the frequency with which FtsA-YFP coincided with a pole in wild type, Δ*minCD*, and Δ*minJ*. For wild type this percentage was only 20% (Fig. 2B). In both Δ*minCD* and Δ*minJ* 70–80% of poles contain FtsA-YFP. It was also found that in the absence of MinCD, all MinJ-CFP signals co-localized with FtsA-YFP at cell poles, however, there are additional FtsA structures at midcell positions without MinJ localization. In contrast in wild type background FtsA and MinJ hardly co-localize (Figure S1). In wild type cells, FtsA-YFP localization is restricted to the midcell while MinJ-CFP is confined to poles and therefore they rarely co-localize. In a Δ*minCD* strain, 84.8% of MinJ-CFP bands were associated with FtsA-YFP, whereas in wild type, this percentage was 20.9%. We took this observation as an indication that in absence of a functional Min system disassembly of the divisomes could be defective, increasing the chance that FtsA and MinJ colocalize.

**Figure 3:**
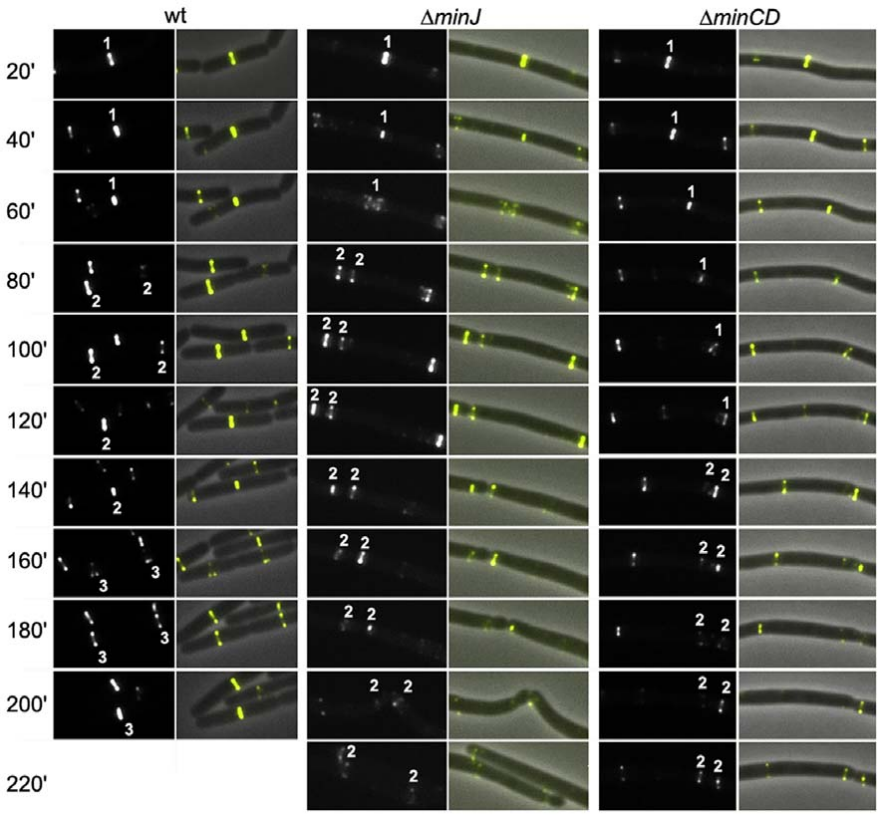
Time-lapse microscopy of FtsA-YFP. On the left, FtsA-YFP in wild type (SB067), center: in Δ*minJ* (SB066) and right in Δ*minCD* (SB060). Numbers on the left indicate minutes and numbers on the FtsA-YFP images show the generation of the rings. In wild type, ring 1 rapidly disappears and new rings are formed at midcell of the two progeny cells (rings 2) which also disappear after division is complete, while new rings again appear at midcell. In the absence of MinJ, the FtsA-YFP ring in this strain (ring 1) is not disassembled and instead begins forming double rings (rings 2). The same was observed for Δ*minCD*. The merge image is an overlay of the phase contrast image with the corresponding FtsA-YFP signal.

The traditional model of the Min system states that in the absence of one of the components, FtsZ-rings, and therefore other components of the divisome, are free to assemble at the poles. However, the microscopy data indicates that FtsA-YFP remains associated with the young poles, instead of re-assembling. To show this, we carried out time lapse microscopy with FtsA-YFP in different backgrounds. In wild type cells, FtsA-YFP forms a band at midcell, which rapidly constricts and disappears. Under the conditions tested, a band of FtsA-YFP is usually present for 60–80 minutes and then rapidly disappears. However, in strain SB066, which is deficient in MinJ and expresses FtsA-YFP, FtsA-YFP rings did not disappear, but rather remained at the cell pole (Fig. 3, Δ*minJ*). In a filamentous Δ*minJ* cell, different FtsA-YFP structures can be visualized, from very bright bands to less intense helical structures. Interestingly, the bright bands were usually the bands that developed into doublets (double rings). This did not seem to be only the case for Δ*minJ* cells, since this was also observed in cells deficient in MinCD (Fig. 3, Δ*minCD*). Also, these results are not due to overexpression of FtsA-YFP, since we did not observe the same in wild type cells expressing FtsA-YFP. It should be noted that the FtsA-YFP structures at the poles in the *min* mutant backgrounds persisted and, hence, we concluded that these structures did not reassemble, but instead never completely disassembled. Thus, these results clearly show that in Min deficient cells, FtsA-YFP is not disassembled, and that the rings can go on to form doublets.

### Late division proteins are retained at the poles in Min-deficient cells

Previously, it was shown that GFP-PBP-2B and GFP-FtsL do not localize to midcell positions in a Δ*minJ* strain [36]. We wanted to analyze whether only a loss of MinJ influences the localization of late division proteins or whether MinD might also play a role in localization of proteins other than FtsZ. Up to now, there has been no evidence that MinCD is involved in PBP-2B binding to the Z-ring.

To this end, we expressed GFP-PBP-2B in Δ*minJ*, Δ*minC*, Δ*minCD*, and Δ*minD* (strains 3122, SB051, SB055, SB054, and, SB053 respectively). In wild type cells, GFP-PBP-2B is present in the membrane and assembles into a ring at midcell (Figs. S2 and S4, GFP-PBP-2B, wt). GFP-PBP-2B regularly forms rings in Δ*minC*, Δ*minD*, and Δ*minCD*. However, the protein fails to form rings in the Δ*minJ* strain except at the rarely formed septa. (Fig. S2, Δ*minJ*). The same experiments were carried out with GFP-FtsL as well, where identical results were obtained (supplemental material Fig. S3). This indicates that membrane proteins of the divisome are not dependent on either MinC or MinD for correct localization, but require MinJ.

It should be noted, however, that GFP-PBP-2B is often found in large concentrations at cell poles in Δ*minC*, Δ*minD*, and Δ*minCD*. Again, it is important to note that the two proteins coincide with the membrane stain, indicating that they are likely not disassembled following division. In wild type cells, the highest concentration of GFP-PBP-2B is at midcell or recently completed septa, although some protein can be found at the cell poles (Fig. S2, wt). However, in Δ*minC*, Δ*minD*, and Δ*minCD* high concentrations of the fusion proteins can be found at the cell poles, in some cases more than at midcell or recently completed septa.

**Figure 4:**
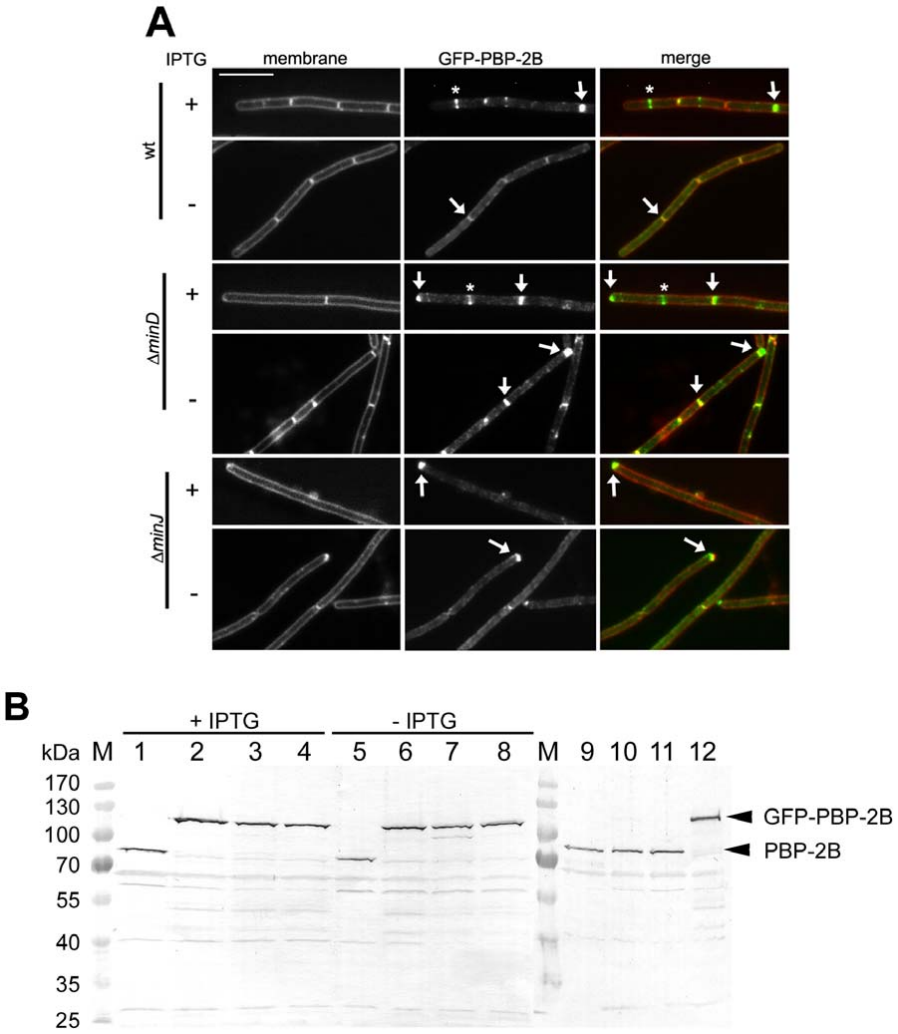
GFP-PBP-2B remains at the cell pole in Δmin cells. **A**. Shown are strains SB088 (GFP-PBP-2B), SB092 (GFP-PBP-2B Δ*minD*), and SB090 (GFP-PBP-2B Δ*minJ*) grown with (+) and without (−) 1 mM IPTG (pre-cultures were grown with 1 mM IPTG). From left to right is shown the membrane stain, GFP-PBP-2B and a merged image. Scale bar is 5 µm. **B**. Western blot of different *ftsZ*/*min* mutants with α-PBP-2B. The loading pattern of the different lanes is as follows: 1/5: FtsZ^+^ (strain 1801), 2/6: FtsZ^+^ GFP-PBP-2B^+^ (strain SB088), 3/7: FtsZ^+^ Δ*minD* GFP-PBP-2B^+^ (strain SB090), 4/8: FtsZ^+^ Δ*minJ* GFP-PBP-2B^+^ (strain SB092), 9: wild type (strain 168), 10: Δ*minJ* (strain RD021), 11: Δ*minDJ* (strain SB075), 12: GFP-PBP-2B (strain 3122). Lanes with molecular mass standard are labelled with M. Induction of GFP-PBP-2B was done with 0.5% xylose and FtsZ expression was induced with 1 mM IPTG (FtsZ^+^) or depleted (FtsZ^−^). Note that a full-length GFP-PBP-2B band is at 106.2 kDA and the native PBP-2B band is seen at 79.1 kDa.

To further corroborate that GFP-PBP-2B does not disassemble but instead remains associated with the poles, we made use of an FtsZ-depletable strain, in which expression of FtsZ can be induced with IPTG. GFP-PBP-2B was expressed in this strain in wild type, (SB088) Δ*minD* (SB092) and Δ*minJ* (SB090). In wild type cells expressing of FtsZ GFP-PBP-2B bands were found at midcell (Fig. 4A, wt +IPTG, asterisk) and only sometimes at the poles (arrow, Fig. 4 A) as described previously, while in the absence of MinD GFP-PBP-2B localized significantly more often at the poles In the absence of MinJ barely any midcell bands were observed but a signal of GFP-PBP-2B was observed at the poles. In cells depleted for FtsZ the difference in wild type and *min* mutant background became even clearer. In the wild type strain, depletion of FtsZ led to a reduction in the signal needed to visualize GFP-PBP-2B, although the protein seemed to be distributed along the membrane, with a slight accumulation at the poles (Fig. 4, wt –IPTG, arrows) In the absence of MinD, GFP-PBP-2B bands were still bright, although as expected, barely any midcell bands could be visualized. Instead, the protein was only found at the poles, forming very bright bands (Fig. 4A, Δ*minD –*IPTG, arrows). In the absence of MinJ, the poles also showed bright bands of GFP-PBP-2B (Fig. 4A, Δ*minJ –*IPTG, arrows). Since FtsZ-depleted cells do not divide as attested by the elongation of these cells, the presence of GFP-PBP-2B at the poles must be due to the failure of the divisome to disassemble, since reassembly does not take place. Thus, the presence of PBP-2B at the cell poles lends support to the notion that the divisome is still assembled at the cell poles in absence of a functional Min system.

Finally, we wanted to test whether we could observe the PBP-2B stabilization in *min* mutants on the protein level. Therefore, we performed immunoblots with several strains expressing native PBP-2B or GFP-PBP-2B and used antibodies against PBP-2B in order to detect the PBP-2B levels. In support with our hypothesis, we found that PBP-2B levels in all our strains were similar to the wild type levels of PBP-2B (Fig. 4 B). Cells expressing GFP-PBP-2B had similar amounts of protein and seemed similarly stable compared to wild type protein. Depletion of FtsZ or loss of MinJ or MinD did not alter the PBP-2B levels (Fig. 4B). We conclude that in our strains the total amount of GFP-PBP-2B is not elevated compared to the native protein and hence localization of GFP-PBP-2B is not due to overexpression artifacts.

**Figure 5:**
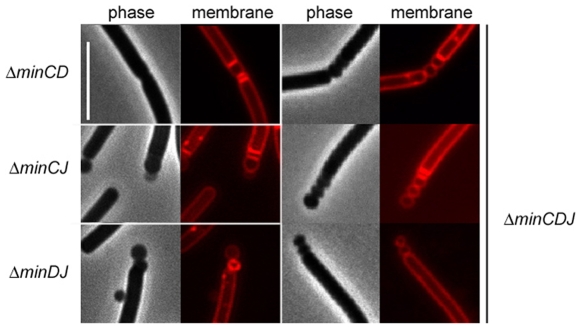
Cells without a functional Min system form multiple minicells. Shown are examples for Δ*minCD* (3309), Δ*minCJ* (SB074), Δ*minDJ* (SB075), and Δ*minCDJ* (MB012), with phase images and membrane stains taken for a few exemplary cells. Note the formation of 2–4 minicells in a row, indicating that a divisome that fails to disassemble often initiates a new round of division, resulting in multiple minicell formation. Scale bar is 5 µm.

The failure to disassemble the divisome after septation leads to minicell formation that could in theory occur more than once at a given site. Indeed we observed consecutive divisions leading to multiple minicells in a row. If minicells would only be formed by aberrant division site selection leading to polar division, only one minicell at each pole should be observed. However, the fact that we find up to four minicells in a row strongly supports the notion that a once assembled divisome keeps on dividing the cell (Fig. 5).

### Analysis of MinJ domains

**Figure 6:**
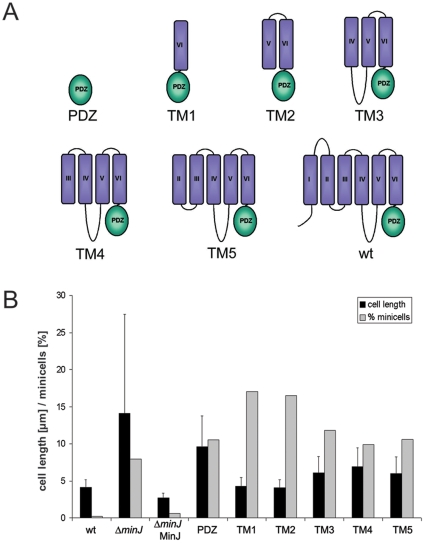
MinJ is able to modulate MinCD activity. **A**. A series of MinJ truncations were created to test which domains are important for function. Note that all truncation were expressed as C-terminal GFP fusions. Localization of the fusion proteins can be found in supplemental material Fig. S5. These truncations include the soluble PDZ domain, TM1, containing the PDZ domain and the last transmembrane helix; TM2, with the PDZ domain and the last two transmembrane helices, and so forth. **B**. To test functionality, they were expressed in Δ*minJ* cells and the cell length and amount of minicells were measured. From left to right, strains tested were wild type (168), Δ*minJ* (RD021), Δ*minJ* MinJ^+^ (MB004), PDZ (SB010), TM1 (SB004), TM2 (SB005), TM3 (SB006), TM4 (SB007) and TM5 (SB008). Grey bars indicate the percentage of minicells produced, and the black bars indicate the average cell length. Expression of TM1 and TM2 led to an identical length as wild type, although they produced even more minicells than the MinJ knockout.

The results shown above provide a strong indication that MinJ and MinD contribute to divisome stability, albeit to different degrees. While MinJ seems to be a central component affecting divisome stability, MinD has only a minor influence. A possible explanation could be that MinD acts through MinJ by regulating MinJ into a state that allows divisome disassembly. If this hypothesis might be true, we should be able to isolate mutations of MinJ that react differentially in the presence of MinCD. To this end, a series of MinJ truncations were constructed. MinJ is a transmembrane protein and predicted to have six transmembrane helices and a cytoplasmic N-terminal tail and a C-terminal PDZ domain, also oriented to the cytoplasm (protease accessibility studies, M. Bramkamp, unpublished). The truncations were systematic in nature, including a soluble PDZ domain, the PDZ domain with one transmembrane helix (TM1), with two transmembrane helices (TM2) etc. (see Fig. 6A). All truncations were expressed as C-terminal translational fusions to GFP. These were expressed in a Δ*minJ* background and the cell length and minicell production was measured to determine their functionality. With the exception of the soluble PDZ domain, all constructs were membrane associated as judged by their GFP-visualized localization (Fig. S5) and immunoblots (data not shown). However, it should be noted that we have not determined the exact topology of the constructs; hence we can only say whether they are membrane associated or soluble. The functional assay *in vivo* was taken as an indication for partial function.

All truncations, when overexpressed in wild type, did not alter the cell length and minicell production (data not shown). When expressed in Δ*minJ*, differential effects could be seen. Interestingly, expression of the PDZ domain alone was able to reduce the cell length although the amount of minicells was not significantly altered (Fig. 6B). However, the protein did not localize to any particular spot in the cell (Fig. S5). Expression of TM3, TM4 and TM5 led to significantly shorter cells, although more minicells were formed than in Δ*minJ*. TM4 did at times form a clear band in the cell; TM5 was often visualized as a spot around midcell (Fig. S5). The most interesting results were obtained when expressing TM1 and TM2. These two proteins were able to completely complement the cell length phenotype of Δ*minJ*, as the cell length was identical to wild type (Fig. 6B). However, strains expressing TM1 and TM2 led to a significant increase in minicell production. This indicates that increased cell division efficiency, which leads to shorter cells, is always at the cost of aberrant cell division at the poles in strains lacking a functional Min system. It also shows that there are MinJ variants which still allow for proper division at midcell, but are unable to prevent division at the poles. It is also important to note that the cell length of TM1 and TM2 is identical to wild type and not to Δ*minCD* mutants, indicating that cell division at midcell proceeds completely normal in these cells. TM1 and TM2 did form relatively clear bands at midcell, indicating that they were able to localize to a certain extent (Fig. S5). Presumably, these truncated versions of MinJ are unable to be regulated by MinD, leading to constant ‘positive’ cell division, even at the poles.

### Overexpression of MinD in the absence of MinJ leads to lethal filamentation

**Figure 7:**
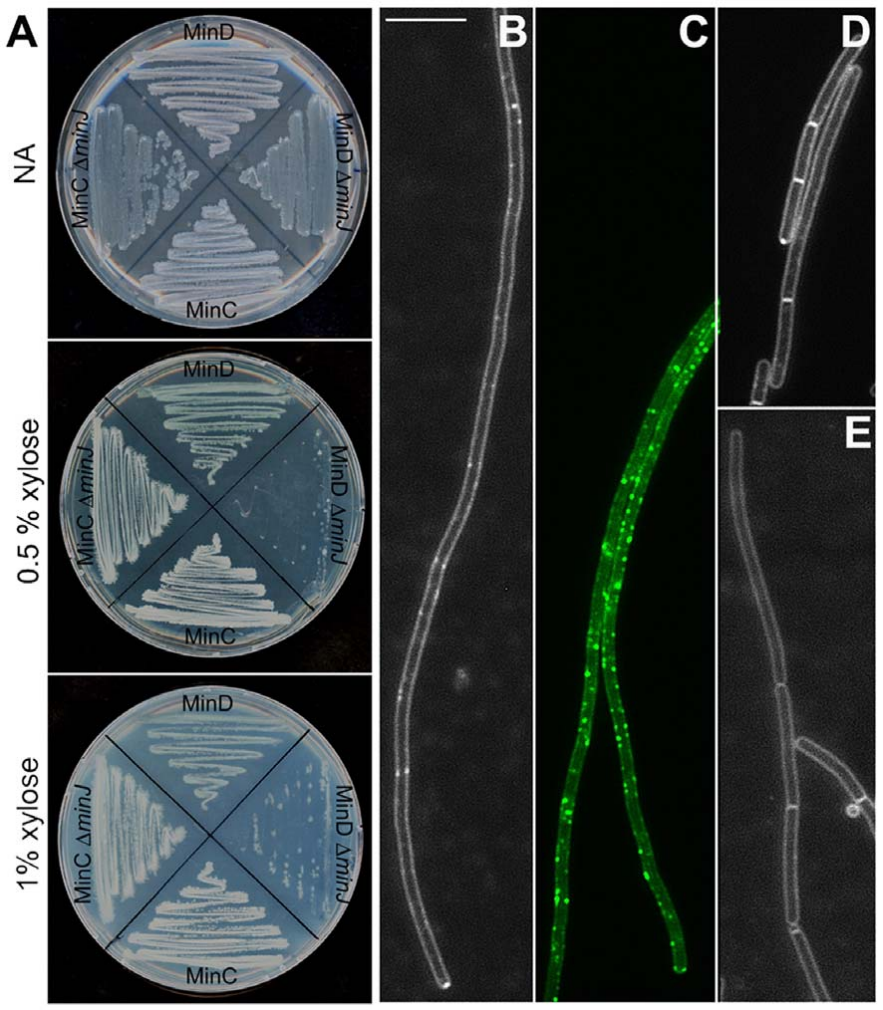
MinD-GFP overexpression increases the cell length. **A**. Nutrient agar plates containing, from top to bottom, 0, 0.5%, and 1% xylose inoculated with strains MinD^+^ (SB076), MinD^+^ Δ*minJ* (SB078), MinC^+^ (SB080), MinC^+^ Δ*minJ* (SB082). Cells overexpressing MinD in Δ*minJ* have a growth defect and grow with difficulty on nutrient agar plates supplemented with 0.5 and 1% xylose. **B**. MinD overexpression in Δ*minJ* (SB078) results in extremely long filamentous cells. **C**. MinD-GFP localizes in foci all over the cell when overexpressed (with 1% xylose) in Δ*minJ* background (SB052). **D**. MinD overexpression in wild type (SB076) leads to weak filamentation, although many cells are of normal length. **E**. MinC overexpression in Δ*minJ* (SB082) does not lead to any increased filamentation (see also Table 1). Scale bar is 5 µm.

Results shown above implied that MinJ and MinD have different effects on the divisome stability and previously published results suggested that MinD and MinJ are antagonistic [36], [37]. In order to address the interaction between MinD and MinJ in more detail, we studied overexpression of MinCD in a *minJ* background. To this end we expressed MinC and MinD ectopically under control of the *P_xyl_* promoter. The resulting strains (MinC, SB080 and MinD, SB076) were subsequently transformed with *minJ::tet* genomic DNA to generate strains SB081 (MinC^+^ Δ*minJ*) and SB077 (MinD^+^ Δ*minJ*). These four strains were then streaked on nutrient agar plates containing no xylose, 0.5% xylose and 1% xylose to induce expression of MinC and MinD. As Fig. 7 shows, overexpression of MinC in wild type cells and Δ*minJ* has no effect on the growth of these strains. Overexpression of MinD in wild type has no effect on growth either. However, the MinD^+^ Δ*minJ* strain has difficulty growing on plates containing 0.5% and 1% xylose. We then analyzed these cells microscopically to determine their morphology. MinD^+^ Δ*minJ* cells are extremely long and filamentous (Fig. 7B and Table 1). We found that the average cell length of these cells was 76.4 µm, which is significantly higher than the average cell length of Δ*minJ* cells (14.1 µm). Overexpression of MinD in wild type cells also leads to filamentation: the average cell length of this strain is 7.3 µm while wild type cells have an average length of 2.8 µm. We also looked at MinC overexpression in Δ*minJ* and found that this had no effect on the cell length of Δ*minJ* (Fig. 7E and Table 1). The average cell length of Δ*minJ* with MinC overexpression is 14.3 µm, which is almost identical to Δ*minJ* cell length (14.1 µm). Consistent with previous observations [39] MinC overexpression in wild type also did not have an effect on cell length.

**Table 1:** Cell length of different *min* mutant strains.

Strain	Average Cell Length [µm]	Standard Deviation [µm]	n
168 (wild type)	2.76	0.69	263
Δ*minJ* (RD021)	14.12	13.30	100
Δ*minCJ* (SB074)	5.03	1.84	269
Δ*minDJ* (SB075)	5.00	1.46	254
MinC (SB080) 1% xylose	3.38	0.82	273
MinC Δ*minJ* (SB082) 1% xylose	14.26	11.15	136
MinC Δ*minD* (SB081) 1% xylose	4.41	1.75	279
MinC Δ*minDJ* (SB083) 1% xylose	5.30	2.02	271
MinD (SB076) 1% xylose	7.27	9.18	271
MinD Δ*minJ* (SB078) 1% xylose	76.37	19.30	16*
MinD Δ*minC* (SB077) 1% xylose	5.29	4.31	359
MinD Δ*minCJ* (SB079) 1% xylose	5.14	1.91	278

The cell length was measured after staining the membrane with the FM4-64 dye.

*The extremely filamentous phenotype of strain SB078 was seen in all cells, for technical reasons we only measured the length of 16 individual cells.

We wanted to check whether the extremely filamentous phenotype of MinD overexpression in a Δ*minJ* background is due to a complete loss of FtsZ polymers or due to an effect downstream of FtsZ assembly. Therefore we first analyzed whether the overproduced MinD would recruit the actual FtsZ inhibitor MinC in a dispersed fashion through the entire cell.

**Figure 8:**
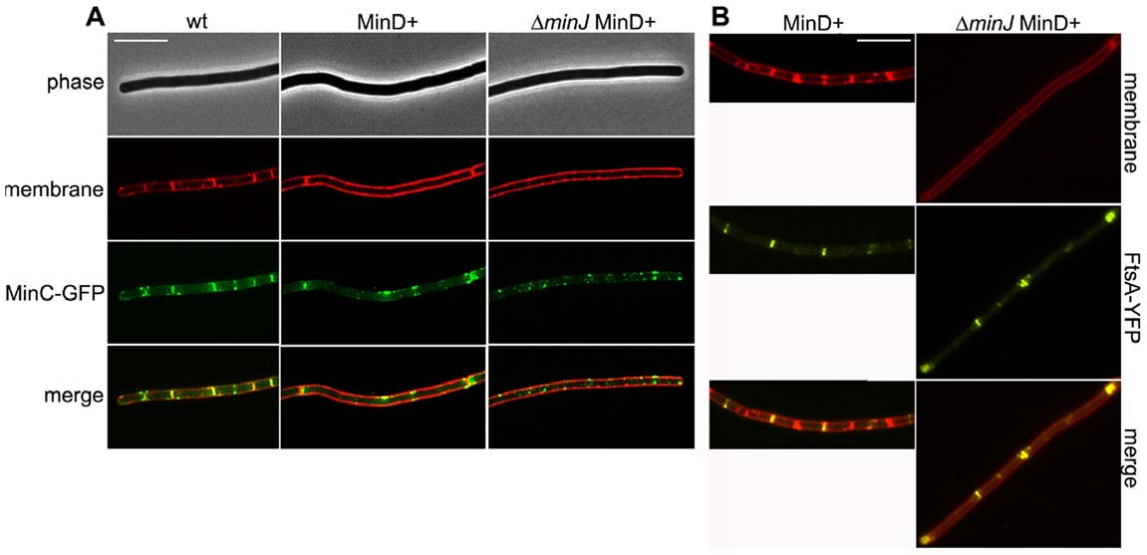
Effects of MinD overexpression on MinC and FtsA. **A**. Left, MinC-GFP localization in wild type (EBS499), center: in MinD^+^ (SB086) induced with 1% xylose, and right, in Δ*minJ* (SB086). top to bottom, phase contrast, membrane stain, MinC-GFP, and a merged image of the membrane stain and MinC-GFP. MinD overexpression leads to a localization pattern of MinC-GFP with multiple rings forming throughout the cell, with double rings frequently being observed. In the absence of MinJ and overexpression of MinD, MinC-GFP becomes completely dispersed and forms foci throughout the cell. **B**. Left: FtsA-YFP localization in MinD^+^ (SB084) and right: in MinD^+^ Δ*minJ* (SB085). In both strains expression of MinD was induced with 1% xylose. From top to bottom, the figure shows an image of the membrane stain (FM4-64), FtsA-YFP localization, and a merged image of FtsA-YFP and the membrane stain. FtsA-YFP expressed in cells overexpressing MinD still localizes in wild type and Δ*minJ* cells indicating that the filamentous cell phenotype must occur downstream of FtsA recruitment to the Z-ring.

To this end, we looked at the localization of MinC-GFP expressed from its native locus in a MinD overexpression strain in the absence and presence of MinJ (SB085 and SB086, respectively). In wild type, MinC-GFP localizes at the poles and at midcell (Fig. 8A, wt). However, in MinD^+^, MinC-GFP can be seen to form multiple bands throughout the cell (Fig. 8A, MinD^+^). Thus, overexpressed MinD sequesters MinC away from the poles. In a strain overexpressing MinD and lacking MinJ, MinC-GFP is dispersed throughout the cell, forming foci, in an identical pattern to GFP-MinD in the absence of MinJ.

Although we have shown that dispersed MinCD does not have an effect on FtsZ-ring formation, but rather on membrane components of the divisome, we wanted to be sure that the filamentous phenotype arising from MinD overexpression in Δ*minJ* is not due to the failure of Z-rings to form or FtsA to localize to the Z-ring. Therefore, we expressed FtsA-YFP in strains SB076 (MinD^+^) and SB078 (Δ*minJ* MinD^+^) and checked localization. As shown in Fig. 8B, FtsA-YFP still localizes (albeit with lower frequency) when MinD is overexpressed in a wild type background as well as a Δ*minJ* background, indicating that the cytosolic components of the divisome are still able to assemble even though MinCD is entirely dispersed throughout the cell. This is indicative of a block in division that lies downstream of FtsZ assembly.

## Discussion

### The Min system contributes to disassembly of the divisome

The classical model of MinCD action states that its activity is restricted to the poles, where minicell formation is most likely to occur. Furthermore, a wealth of data suggests that the Min system acts directly at the level of FtsZ polymerization [19], [40]. However, recently it was shown that the highest chance of minicell formation actually occurs at recently completed division sites, and not at the old poles, challenging the role of the Min system [35]. The preferential localization of *B. subtilis* MinC [35] and MinJ ([36], [37] and this work) to new division sites supports this role. These recent data indicate that the division sites are the most important places for MinCDJ action. In this paper, we show that the main reason for this localization is because at this site the Min system might contribute to the disassembly of the divisome. We have shown that in the absence of one of the components of the Min system, cell division proteins fail to disassemble and remain associated with the new pole. In the absence of MinCD and MinJ, FtsA-YFP, GFP-FtsL and GFP-PBP-2B remain associated with the division site, in contrast to wild type, where these proteins are usually found at midcell. This data also shows that an important determinant in minicell formation is the failure to disassemble the divisome, in which the Min system also plays a role. This failure to disassemble allows cell division proteins to initiate another round of cytokinesis close to the original cell division site. This effect is best seen in a triple Δ*minCDJ* knockout, where it is quite common to see three minicells in a row, indicating that the retained divisome can easily initiate new rounds of cell division (Fig. 5). A similar observation has been made long ago for the MinCDE system in *E. coli* [41]. A combination of a thermosensitive FtsZ variant (*ftsZ84*) and a deletion of the *minCDE* operon resulted in an increased thermosensitivity. Furthermore, in this strain background a high degree of polar divisions was observed, leading to consecutive minicelling. A close inspection using immunofluorescence of the FtsZ polymer structures at the cell poles revealed that the septa were elongated (wider than normal) which was interpreted as indication for a defect in disassembly of the divisomes [41]. Using a FtsA-YFP fusion, we were able to see similar elongated septa in *Bacillus*. However, the higher resolution compared with the earlier immunofluorescence used in the *E. coli* experiments enabled is to identify the elongated septa as spirals that originated from ongoing septations. Thus, MinJ in *B. subtilis* has, at least in part, an analogous role to MinE in *E. coli* in that it regulates the activity of MinCD.

However, it remains difficult to differentiate between reduction in divisome disassembly and immediate reassembly of components close to a used division site. The best argument that the Min system is involved in divisome disassembly stems from the fact that in a *minJ* mutant background the septa at the used sites of septation are spiral-like (or elongated, as it was described in *E. coli* [41]. Possibly, constriction of the cytokinetic ring occurs in a spiral, or diaphragm-like manner. This mechanism would not need constant removal of subunits from the divisome to achieve constriction. In fact the Z-ring is composed of multiple shorter polymers that are associated laterally. Such a construction would make steady-going constriction with removal of individual subunits rather difficult to organize in comparison of a smooth, diaphragm-like constriction. Therefore, the action of MinC might occur in another way than inhibiting FtsZ polymerization. This has in fact been shown both *in vitro* and *in vivo*. *In vitro*, purified MinC did not inhibit FtsZ polymerization significantly, and even in the presence of MinC, FtsZ polymers could be observed using electron microscopy [24], although they were shorter than those incubated without MinC. *In vivo* it was shown that expression of a mutant FtsZ that was predicted to stabilize the polymer could overcome the effects of MinCD overexpression [42]. Both of these data argue that the effect of MinC on FtsZ is not on FtsZ polymerization, but rather, between lateral interactions between FtsZ polymers. The *E. coli* C-terminal MinC together with MinD has been shown to displace FtsA from the Z-ring, which provides an alternative to preventing Z-ring formation by preventing polymerization [43]. However, it is important to note that FtsA is essential for cell division in *E. coli*, while in *B. subtilis*, it is not. Additionally, we have shown that FtsA-YFP still forms rings in cells lacking MinJ, in which MinCD is dispersed, arguing against *B. subtilis* MinC participating in such a displacing function. We think it is possible that MinC, instead of preventing formation of the Z ring, could destabilize the Z ring by interfering with lateral interactions, and in this way aid in the disassembly of the divisome. However, this action of MinCD would require the relay of information regarding the status of division. The data from the MinJ truncation experiments suggest that MinJ may be responsible for this, because a membrane associated MinJ-PDZ domain is able to promote cytokinesis, but is defective in disassembly as judged by the high amount of minicells.

With all the results taken together, we propose a model on the main function of the Min system. In a non-dividing cell, MinCDJ are localized to the cell poles through polar targeting by DivIVA. As the cell grows and the nucleoids are replicated and segregated, a cytokinetic ring is formed at midcell. When the cytokinetic ring is fully formed and the cell is committed to cell division, MinCDJ moves from the poles to the cytokinetic ring, probably following DivIVA which binds to the curved membrane at the inward growing septum [44]. Cell division initiates the formation of a septum, after which MinCDJ promotes the disassembly of the divisome. After this, MinCDJ localize again to the old poles as well as the new poles, and the cycle starts again. In the absence of a functional Min system, a cytokinetic ring is formed between segregated nucleoids, initiating the formation of a septum. However, the cytokinetic ring does not disassemble and remains in close proximity to recently used division sites. As the cell grows and elongates, the cytokinetic ring adjacent to the old septum can initiate a new round of replication, leading to the formation of a minicell. Therefore, the main function of the Min system is to ensure a single round of division per cell cycle by preventing minicell formation through promoting the disassembly of the cytokinetic ring.

## Materials and Methods

### Bacterial strains, plasmids and oligonucleotides

All bacterial strains, plasmids and oligonucleotides are listed in Tables S1, S2, S3, respectively. Strain construction was done using routine protocols. Liquid cultures of *B. subtilis* were grown in MD medium, a modified version of Spizizen Minimal Medium [45]. MD medium contains 10.7 mg ml^−1^ K_2_HPO_4_, 6 mg ml^−1^ KH_2_PO_4_, 1 mg ml^−1^ Na_3_ citrate, 20 mg ml^−1^ glucose, 0.05 20 mg ml^−1^ L-tryptophan, 20 mg ml^−1^ ferric ammonium citrate, 25 mg ml^−1^ L-aspartate, and 0.36 mg ml^−1^ MgSO_4_. MD medium was further supplemented with 1 mg ml^−1^ casamino acids.

Transformations were plated on nutrient agar plates (Oxoid) supplemented with antibiotics as required [5 µg ml^−1^ chloramphenicol, 5 µg ml^−1^ kanamycin, 50 µg ml^−1^ spectinomycin, 0.3 µg ml^−1^ erythromycin, 12 µg ml^−1^ tetracycline]. For experiments requiring induction, medium was supplemented with 1 mM isopropyl-β-D-thiogalactopyronoside (IPTG) or 50 µg ml^−1^ xylose, unless otherwise stated.

### SDS-PAGE and Immunoblotting

SDS-polyacrylamide gelelectrophoresis has been carried out according to a protocol decribed by Laemmli [46]. Samples were subjected to a 10% SDS gel (it should be noted that the samples were not heat denatured prior loading to avoid breakdown of GFP-PBP-2B) and blotted onto a PVF membrane. The blot was incubated with the α-PBP2B (1∶5000) at 4°C for at least 1 h. The blot was then washed with sodium phosphate buffer and incubated with the secondary antibody, anti-rabbit conjugated with alkaline phosphatase (1∶10,000) at 4°C for at least 1 h. The blot was again washed with sodium phosphate buffer and developed with NBT/BCIP.

### Microsopic imaging

For membrane staining a 100 µl culture sample was mixed with 1 µl 1 mM FM®4-64 dye (Invitrogen). Images were taken on a Zeiss AxioImager M1 equipped with a Zeiss AxioCam HRm camera. Generally, an EC Plan-Neofluar 100x, 1.3 Oil Ph3 objective was used. Digital images were acquired with the AxioVision (Zeiss) software and analyzed using the Axiovision 4.6 software (Zeiss). Final image preparation was done in Adobe Photoshop 6.0 (Adobe Systems Incorporated).

### Time lapse microscopy

Time lapse microscopy of GFP-MinJ was carried out as described before [47]. Cells were grown overnight in liquid minimal medium (MM) at 30°C and continuously shaken at 200 rpm. MM contained 62 mM K_2_HPO_4_, 44 mM KH_2_PO_4_, 15 mM (NH_4_)_2_SO_4_, 6.5 mM sodium citrate, 0.8 mM MgSO_4_, 0.02% casamino acids, 27.8 mM glucose, and 0.1 mM L-tryptophan. The pH was set to 7 using a KOH solution. After overnight growth cells were diluted 1∶10 in liquid chemically defined medium (CDM). CDM is a MM solution, but without casamino acids, containing 2.2 mM glucose, 2.1 mM L-glutamic acid, 6 µM L-tryptophan, 7.5 µM MnCl_2_, and 0.15× metal (MT) mix [47]. This CDM was then diluted to 15% before use. Exponentially growing cells were inoculated onto a thin semisolid matrix of low melting point agarose attached to a microscope slide. The slides were prepared using a 125 ml Gene Frame (AB-0578; ABgene) that was attached to a standard microscope slide (CML). The resulting cavity was filled with heated CDM supplemented with 1.5% low-melting-point agarose (A4718; Sigma-Aldrich) and covered with a standard microscope slide. After cooling and removal of the cover slide, strips of CDM-agarose were removed, resulting in a small strip of CDM-agarose (∼1.5 mm wide) in the center of the Gene Frame. This provides air cavities that are essential for efficient growth and spore formation. Cells were spotted onto the strip, and the Gene Frame was sealed with a coverslip (24×60 mm; Menzel GmbH). The microscopy was then carried out on a DeltaVision microscope.

Time-lapse microscopy with FtsA-YFP was carried out using the Zeiss AxioImager M1 equipped with a Zeiss AxioCam HRm camera and using the AxioVision 4.6 software (Carl Zeiss). Cells were grown overnight in MD medium with casamino acids and, the next day, diluted 1∶10 in fresh MD medium supplemented with casamino acids and 1 mM IPTG to induce expression of FtsA-YFP. Slides were prepared as above, but instead of using CDM, MD medium supplemented with casamino acids and 1 mM IPTG was used. After three hours of growth, cells were mounted on slides as described for the above time-lapse microscopy and left to grow at room temperature for about 2 hours. Following this growth on slides, images were taken every 20 minutes for 4 hours.

### 3D reconstruction

A 3D reconstruction of Z-rings as shown in Movie S1 was performed as described before [36].

## Supporting Information

Figure S1 MinJ and FtsA co-localize in Δ*minCD* mutant background. Localization of FtsA-YFP and MinJ-CFP in wild type (SB026) on the left, and Δ*minCD* (SB062) are shown on the right. From top to bottom the image shows the phase contrast, membrane stain, FtsA-YFP, MinJ-CFP and the merged image of the membrane stain, FtsA-YFP and MinJ-CFP. Scale bar is 5 µm.
(1.05 MB TIF)

Figure S2 Late division proteins are retained at the poles in Min-deficient cells. GFP-PBP-2B localization, from top to bottom, in wild type (3122) Δ*minC* (SB055), Δ*minD* (SB053), Δ*minCD* (SB054), Δ*minJ* (SB051), and Δ*minCDJ* (SB065). From left to right, the figure shows phase contrast, membrane stain, GFP-PBP-2B, and a merged image of the membrane stain and GFP-PBP-2B. PBP-2B localizes mostly to midcell, but in cells deficient in MinC or MinD, GFP-PBP-2B is often found at the poles. In a MinJ knockout, GFP-PBP-2B does not localize. However, simultaneous depletion of MinCD results in localization of GFP-PBP-2B to midcell, although it is also retained at the poles. Arrows point exemplarily to a cell pole. Scale bars are 5 µm.
(4.92 MB TIF)

Figure S3 FtsL is retained at the cell poles in absence of the Min system. Shown is the localization of GFP-FtsL in (from top to bottom) wild type (2012), Δ*minC* (SB059), Δ*minD* (SB057), Δ*minCD* (SB058), Δ*minJ* (SB056), and Δ*minCDJ* (SB064). From left to right, the figure shows phase contrast, membrane stain, GFP-FtsL, and a merged image of the membrane stain and GFP-FtsL. Arrows point exemplarily to a cell pole. Scale bars are 5 µm.
(4.32 MB TIF)

Figure S4 Localization of PBP-2B and FtsL in *minCJ* and *minDJ* mutants. GFP-PBP-2B localization in wildtype (3122), Δ*minCJ* (SB070) and Δ*minDJ* (SB071) Bottom: GFP-FtsL localization in wildtype (2012), Δ*minCJ* (SB073), and Δ*minDJ* (SB072). Scale bars are 5 µm. Both GFP-PBP-2B and GFP-FtsL localize in a Δ*minCJ* strain, indicating that dispersed MinD alone cannot inhibit the divisome from forming.
(4.50 MB TIF)

Figure S5 Subcellular localization of MinJ truncations. Localization of different truncations in a Δ*minJ* background. The image shows phase contrast images on top and the corresponding GFP fluorescence in the lower panel. From left to right, the localization of wt (SB002), PDZ (SB018), TM1 (SB012), TM2 (SB013), TM3 (SB014), TM4 (SB015), and TM5 (SB016). Scale bar is 5 µm.
(5.45 MB TIF)

Table S1 Bacterial strains.
(0.13 MB DOC)

Table S2 Plasmids.
(0.04 MB DOC)

Table S3 Oligonucleotides.
(0.03 MB DOC)

Movie S1 Multiple FtsZ-GFP rings in a Δ*minJ* backound. Shown is a 3D reconstruction of multiple Z-rings in a strain expressing FtsZ-GFP lacking MinJ (strain 3869). Note that only one FtsZ ring constricts (the one which has no obvious central hole), while the other rings remain open.
(0.33 MB MPG)

Movie S2 Time lapse microscopy of GFP-MinJ (strain MB001). Cells were grown and analyzed as described (see materials and methods). Phase contrast and deconvolved GFP-MinJ fluorescence are merged. Cells were induced with 0.1% xylose. Still images of this movie are shown in Fig. 1B.
(2.53 MB MPG)
