# Supplementary figures and images for: The MinCDJ System in Bacillus subtilis Prevents Minicell Formation by Promoting Divisome Disassembly
Source: PLoS One. 2010 Mar 24;5(3):e9850. doi: 10.1371/journal.pone.0009850 (PMC2844427; doi:10.1371/journal.pone.0009850)

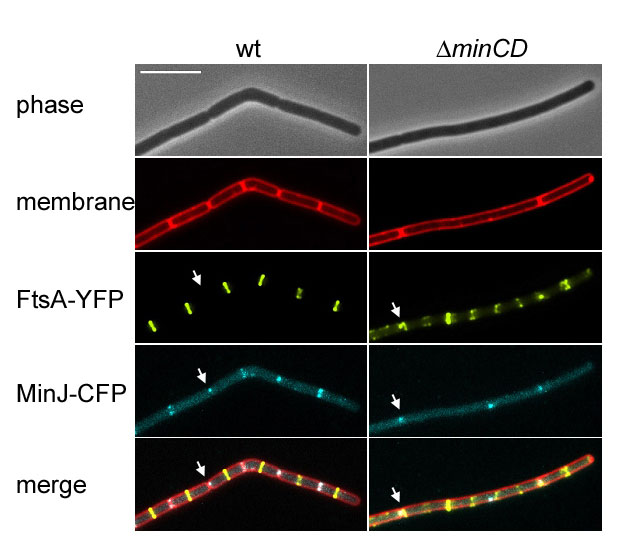

Supplement: Figure S1 — MinJ and FtsA co-localize in ΔminCD mutant background. Localization of FtsA-YFP and MinJ-CFP in wild type (SB026) on the left, and ΔminCD (SB062) are shown on the right. From top to bottom the image shows the phase contrast, membrane stain, FtsA-YFP, MinJ-CFP and the merged image of the membrane stain, FtsA-YFP and MinJ-CFP. Scale bar is 5 µm. (1.05 MB TIF) [file pone.0009850.s001.tif]

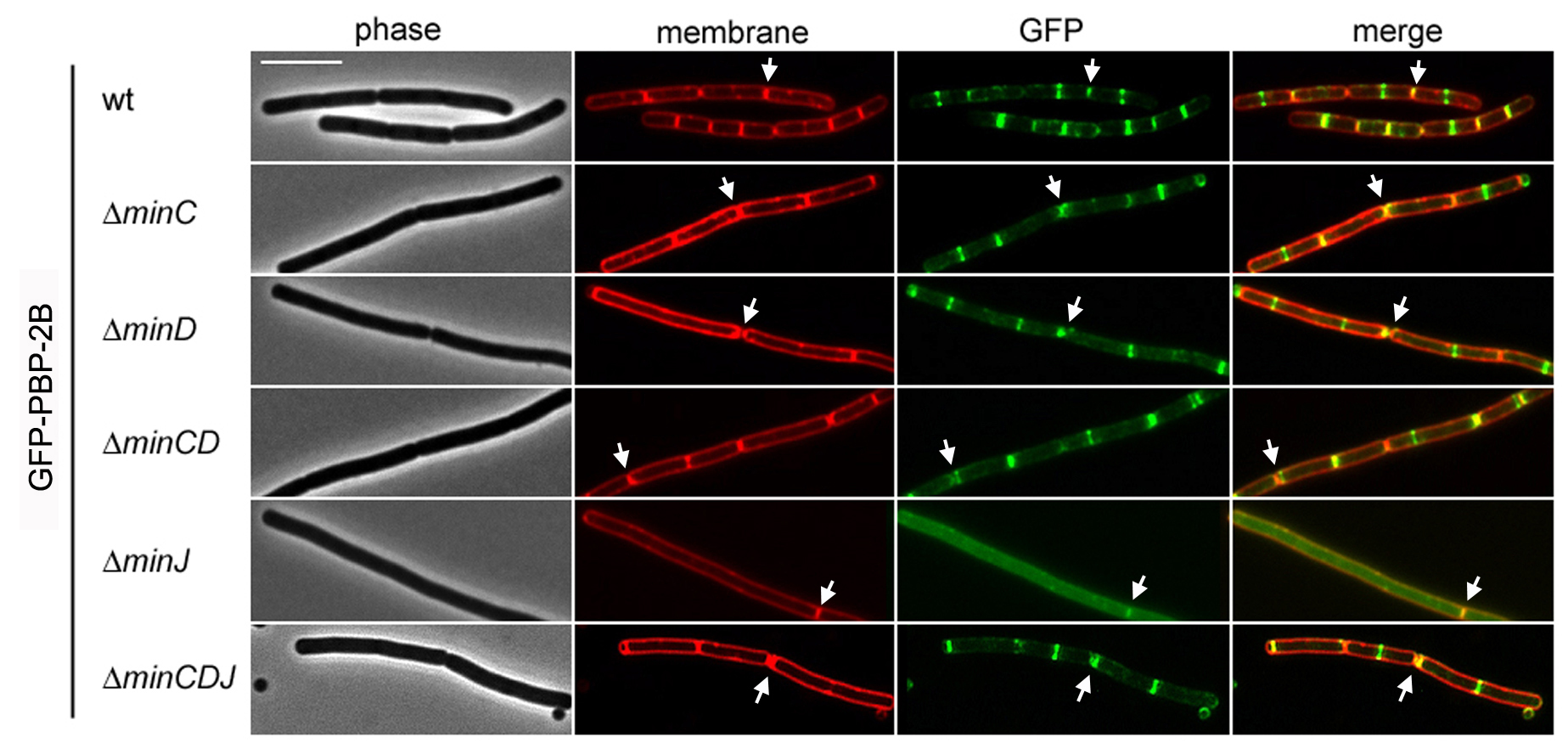

Supplement: Figure S2 — Late division proteins are retained at the poles in Min-deficient cells. GFP-PBP-2B localization, from top to bottom, in wild type (3122) ΔminC (SB055), ΔminD (SB053), ΔminCD (SB054), ΔminJ (SB051), and ΔminCDJ (SB065). From left to right, the figure shows phase contrast, membrane stain, GFP-PBP-2B, and a merged image of the membrane stain and GFP-PBP-2B. PBP-2B localizes mostly to midcell, but in cells deficient in MinC or MinD, GFP-PBP-2B is often found at the poles. In a MinJ knockout, GFP-PBP-2B does not localize. However, simultaneous depletion of MinCD results in localization of GFP-PBP-2B to midcell, although it is also retained at the poles. Arrows point exemplarily to a cell pole. Scale bars are 5 µm. (4.92 MB TIF) [file pone.0009850.s002.tif]

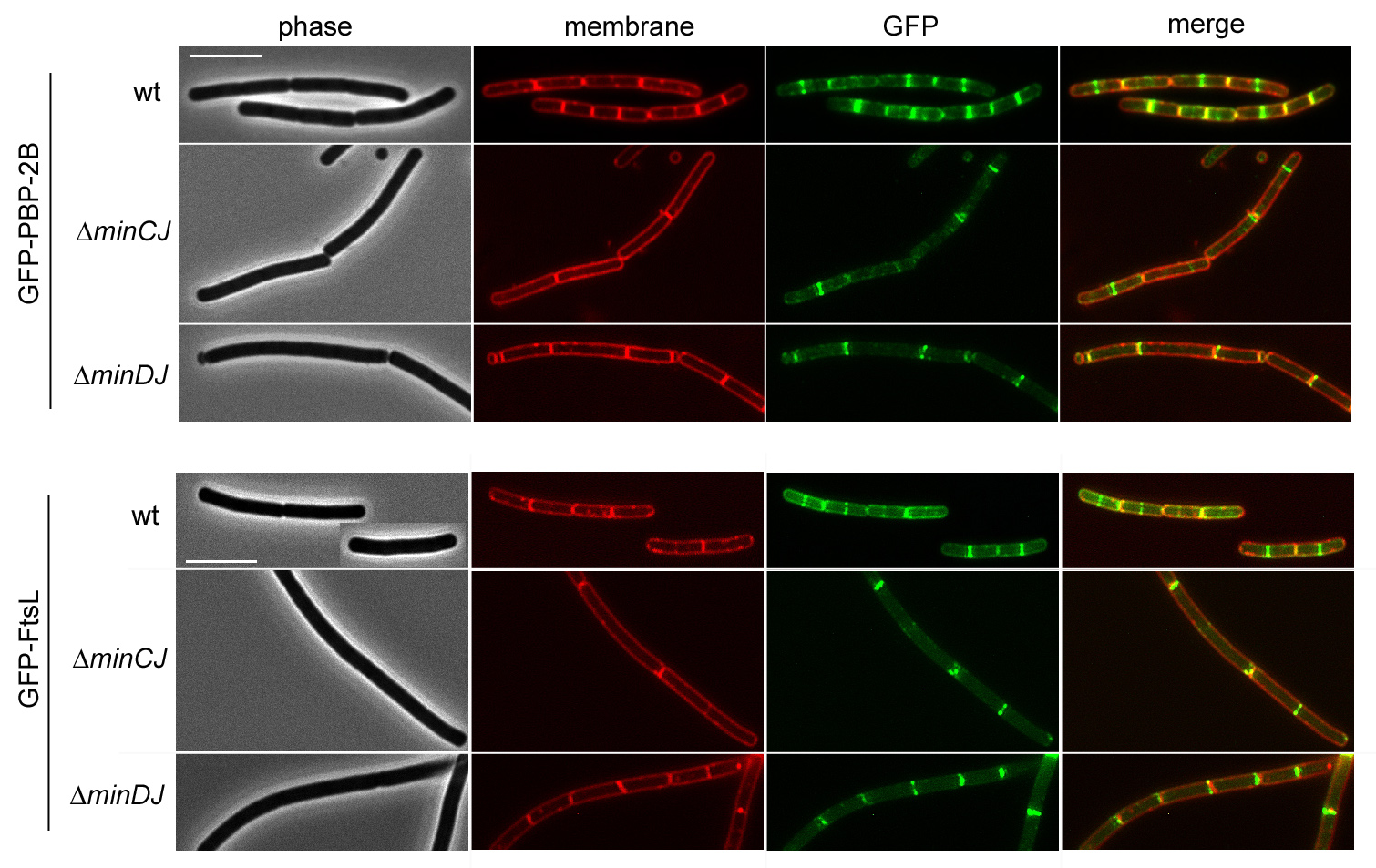

Supplement: Figure S3 — FtsL is retained at the cell poles in absence of the Min system. Shown is the localization of GFP-FtsL in (from top to bottom) wild type (2012), ΔminC (SB059), ΔminD (SB057), ΔminCD (SB058), ΔminJ (SB056), and ΔminCDJ (SB064). From left to right, the figure shows phase contrast, membrane stain, GFP-FtsL, and a merged image of the membrane stain and GFP-FtsL. Arrows point exemplarily to a cell pole. Scale bars are 5 µm. (4.32 MB TIF) [file pone.0009850.s003.tif]

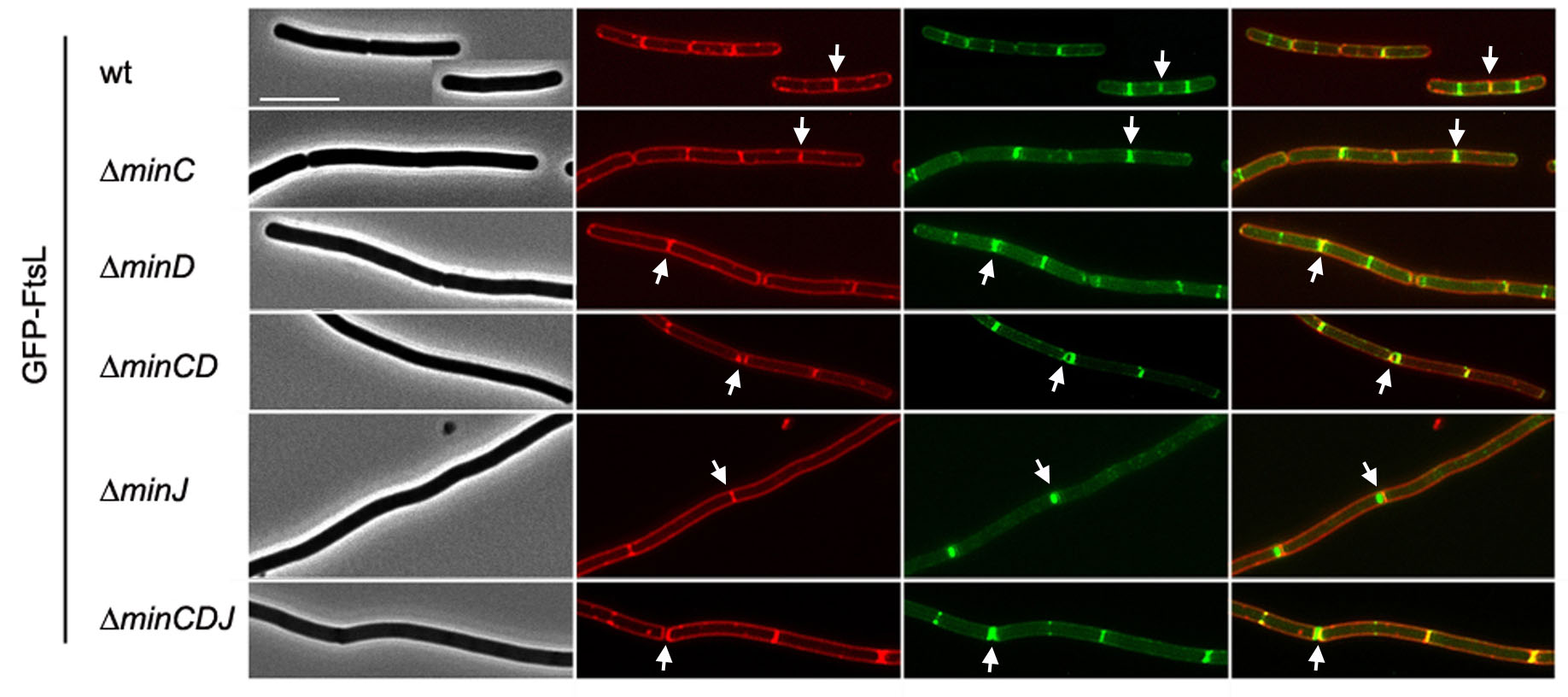

Supplement: Figure S4 — Localization of PBP-2B and FtsL in minCJ and minDJ mutants. GFP-PBP-2B localization in wildtype (3122), ΔminCJ (SB070) and ΔminDJ (SB071) Bottom: GFP-FtsL localization in wildtype (2012), ΔminCJ (SB073), and ΔminDJ (SB072). Scale bars are 5 µm. Both GFP-PBP-2B and GFP-FtsL localize in a ΔminCJ strain, indicating that dispersed MinD alone cannot inhibit the divisome from forming. (4.50 MB TIF) [file pone.0009850.s004.tif]

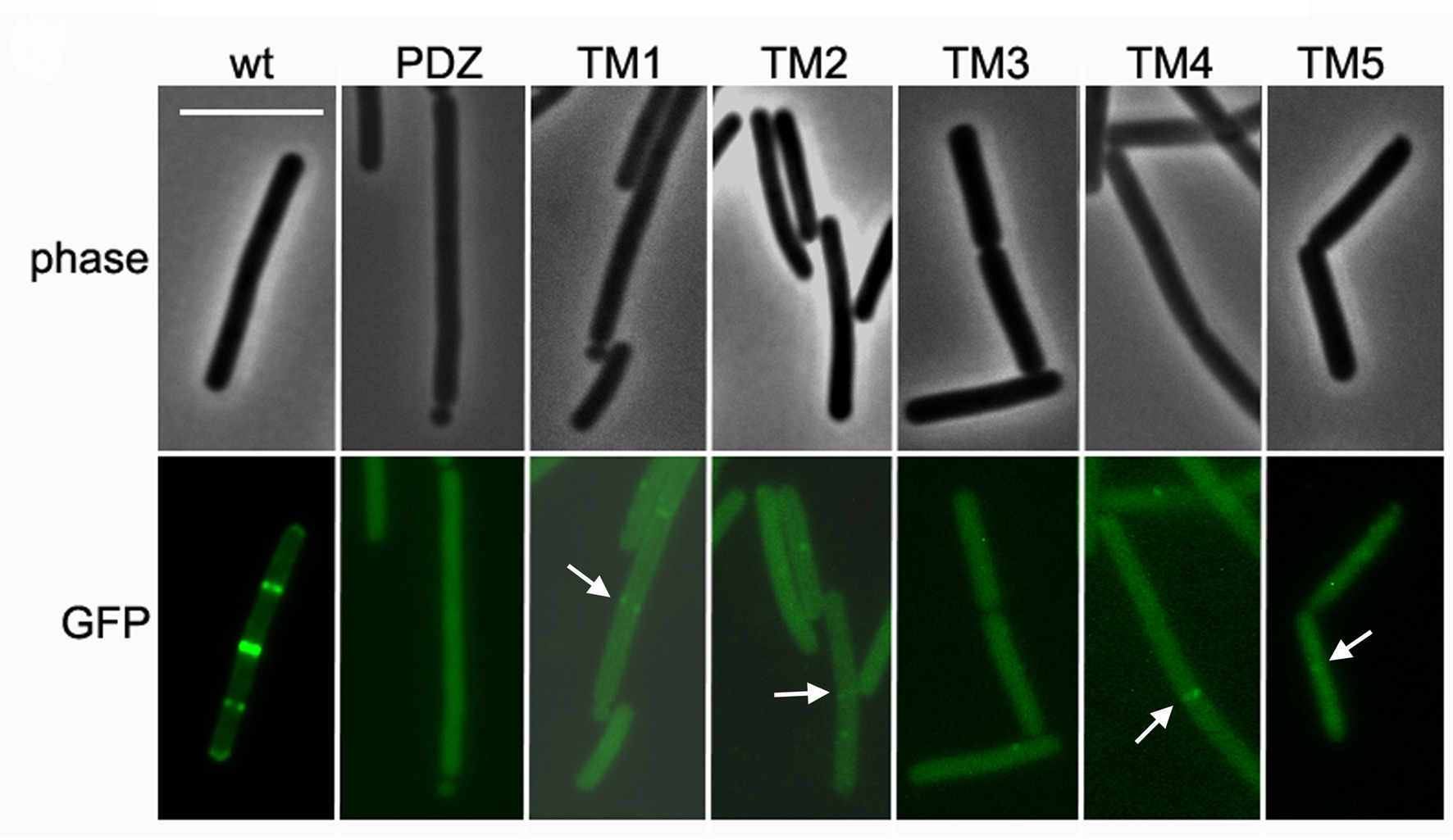

Supplement: Figure S5 — Subcellular localization of MinJ truncations. Localization of different truncations in a ΔminJ background. The image shows phase contrast images on top and the corresponding GFP fluorescence in the lower panel. From left to right, the localization of wt (SB002), PDZ (SB018), TM1 (SB012), TM2 (SB013), TM3 (SB014), TM4 (SB015), and TM5 (SB016). Scale bar is 5 µm. (5.45 MB TIF) [file pone.0009850.s005.tif]
