# Supplementary material for: The MinCDJ System in Bacillus subtilis Prevents Minicell Formation by Promoting Divisome Disassembly
Source: PLoS One. 2010 Mar 24;5(3):e9850. doi: 10.1371/journal.pone.0009850 (PMC2844427; doi:10.1371/journal.pone.0009850)
Supplement: Table S1 — Bacterial strains. (0.13 MB DOC) [file pone.0009850.s006.doc]

**Table S1: Bacterial strains**

| **Strain** | **Relevant characteristics** | **Source/construction** |
| --- | --- | --- |
| 168 | *trpC2* | Laboratory stock |
| RD021 | *trpC2 yvjD::tet* | [1] |
| MB012 | *trpC2 yvjD::tet minCD::aph-A3* | [1] |
| MB001 | *trpC2* (*amyE*::*spc* *P_xyl_ gfp*-*yvjD*) | [1] |
| MB004 | *trpC2* (*amyE*::*spc* *P_xyl_* *yvjD*-*gfp*) *yvjD*::pMUTIN4 | [1] |
| SB002 | *trpC2 yvjD::tet (amyE::spec P_xyl_ yvjD-gfp)* | MB002 transformed with RD021 |
| SB003 | *trpC2 yvjD::yvjD _pSG1186* | 168 transformed with pSB001 |
| SB004 | *trpC2 (amyE::spec P_xyl_ yvjd Δ243-gfp)* | 168 transformed with pSB010 |
| SB005 | *trpC2 (amyE::spec P_xyl_ yvjd Δ200-gfp)* | 168 transformed with pSB011 |
| SB006 | *trpC2 (amyE::spec P_xyl_ yvjd Δ130-gfp)* | 168 transformed with pSB012 |
| SB007 | *trpC2 (amyE::spec P_xyl_ yvjd Δ97-gfp)* | 168 transformed with pSB013 |
| SB008 | *trpC2 (amyE::spec P_xyl_ yvjd Δ57-gfp)* | 168 transformed with pSB014 |
| SB010 | *trpC2 (amyE::spec P_xyl_ yvjd Δ278-gfp)* | 168 transformed with pSB016 |
| SB012 | *trpC2 yvjD::tet (amyE::spec P_xyl_ yvjd Δ243-gfp)* | SB004 transformed with RD021 |
| SB013 | *trpC2 yvjD::tet (amyE::spec P_xyl_ yvjd Δ200-gfp)* | SB005 transformed with RD021 |
| SB014 | *trpC2 yvjD::tet (amyE::spec P_xyl_ yvjd Δ130-gfp)* | SB006 transformed with RD021 |
| SB015 | *trpC2 yvjD::tet (amyE::spec P_xyl_ yvjd Δ97-gfp)* | SB007 transformed with RD021 |
| SB016 | *trpC2 yvjD::tet (amyE::spec P_xyl_ yvjd Δ57-gfp)* | SB008 transformed with RD021 |
| SB018 | *trpC2 yvjD::tet (amyE::spec P_xyl_ yvjd Δ278-gfp)* | SB010 transformed with RD021 |
| YK20 | CRK6000 *(aprE::spec P_spac_ yfp-ftsA)* | [2] |
| SB026 | *trpC2* *(aprE::spec P_spac_ yfp-ftsA) yvjD::yvjD_pSG1186* | Strain YK20 transformed into SB003 [1] |
| 3309 | *trpC2 minCD::aph-A3* | Leendert Hamoen |
| 3381 | *trpC2 minC::aph-A3* | Leendert Hamoen |
| 3869 | *trpC2* (*amyE*::*spc* *Pxyl gfp-ftsZ*) *yvjD*::pMUTIN4 | [1] |
| SG1901 | *trpC2* *minD::erm* | [3] |
| 3122 | *trpC2 pbpB::pSG5061 (cat P_xyl_-gfp-pbpB^1−825^)* | [4] |
| SB051 | *trpC2 minJ ::tet*  *pbpB::pSG5061 (cat P_xyl_-gfp-pbpB^1−825^)* | 3122 transformed with RD021 |
| SB053 | *trpC2 minD::erm pbpB::pSG5061 (cat P_xyl_-gfp-pbpB^1−825^)* | 3122 transformed with SG1901 |
| SB054 | *trpC2 minCD::aph-A3 pbpB::pSG5061 (cat P_xyl_-gfp-pbpB^1−825^)* | 3122 transformed with 3309 |
| SB055 | *trpC2 minC::aph-A3 pbpB::pSG5061 (cat P_xyl_-gfp-pbpB^1−825^)* | 3122 transformed with 3381 |
| 2012 | *trpC2* (*amyE*::*spc* *P_xyl_ gfp*-*ftsL*) | [5] |
| SB056 | *trpC2 yvjD::tet amyE::spec P_xyl_ gfp-ftsL* | 2012 transformed with RD021 |
| SB057 | *trpC2 minD::erm amyE::spec P_xyl_ gfp-ftsL* | 2012 transformed with SG1901 |
| SB058 | *trpC2 minCD::aph-A3 amyE::spec P_xyl_ gfp-ftsL* | 2012 transformed with 3309 |
| SB059 | *trpC2 minC::aph-A3 amyE::spec P_xyl_ gfp-ftsL* | 2012 transformed with 3381 |
| SB050 | *trpC2* *minD::erm yvjD::yvjD_pSG1186* | SG1901 transformed with SB003 |
| 4041 | *trpC2 divIVA::tet* | Leendert Hamoen |
| SB060 | *trpC2 minCD::aph-A3 aprE::spec P_spac_ yfp-ftsA* | YK20 transformed with 3309 |
| SB061 | *trpC2 minCD::aph-A3 minJ::tet aprE::spec P_spac_ yfp-ftsA* | SB060 transformed with RD021 |
| SB062 | *trpC2 minCD::aph-A3 yvjD::yvjD_pSG1186 aprE::spec P_spac_ yfp-ftsA* | SB026 transformed with 3309 |
| SB064 | *trpC2 minCD::aph-A3 ftsL::neo amyE::spec P_xyl_ gfp-ftsL yvjD::tet* | SB058 transformed with RD021 |
| SB065 | *trpC2 minCD::aph-A3 pbpB::pSG5061 (cat P_xyl_-gfp-pbpB^1−825^) yvjD::tet* | SB054 transformed with RD021 |
| SB066 | *trpC2 yvjD::tet aprE::spec P_spac_ yfp-ftsA* | RD021 transformed with YK20 |
| SB067 | *trpC2 aprE::spec P_spac_ yfp-ftsA* | 168 transformed with YK20 |
| SB068 | *trpC2 (amyE::spec P_xyl_ gfp-minD) yvjD::tet* | MB005 transformed with RD021 |
| SB069 | *trpC2 (amyE::spec P_xyl_ gfp-minD) minC::kan yvjD::tet* | SB068 transformed with 3381 |
| SB070 | *trpC2 minC::aph-A3 pbpB::pSG5061 (cat P_xyl_-gfp-pbpB^1−825^) yvjD::tet* | SB055 transformed with RD021 |
| SB071 | *trpC2 minD::erm pbpB::pSG5061 (cat P_xyl_-gfp-pbpB^1−825^) yvjD::tet* | SB053 transformed with RD021 |
| SB072 | *trpC2 minD::erm yvjD::tet amyE::spec P_xyl_ gfp-ftsL* | SB057 transformed with RD021 |
| SB073 | *trpC2 minC::aph-A3yvjD::tet amyE::spec P_xyl_ gfp-ftsL* | SB059 transformed with RD021 |
| SB074 | *trpC2 minC::aph-A3 yvjD::tet* | 3381 transformed with RD021 |
| SB075 | *trpC2 minD::erm yvjD::tet* | SG091 transformed with RD021 |
| SB076 | *trpC2 (amyE::cam P_xyl_ minD)* | 168 transformed with plasmid pSB025 |
| SB077 | *trpC2 (amyE::cam P_xyl_ minD) minC::kan* | SB076 transformed with 3309 |
| SB078 | *trpC2 (amyE::cam P_xyl_ minD) yvjD::tet* | SB076 transformed with RD021 |
| SB079 | *trpC2 (amyE::cam P_xyl_ minD) minC::kan yvjD::tet* | SB077 transformed with RD021 |
| SB080 | *trpC2 (amyE::cam P_xyl_ minC)* | 168 transformed with plasmid pSB024 |
| SB081 | *trpC2 (amyE::cam P_xyl_ minC) minD::erm* | SB080 transformed with SB1901 |
| SB082 | *trpC2 (amyE::cam P_xyl_ minC) yvjD::tet* | SB080 transformed with RD021 |
| SB083 | *trpC2 (amyE::cam P_xyl_ minC) minD::erm yvjD::tet* | SB081 transformed with RD021 |
| SB084 | *trpC2 (amyE::cam P_xyl_ minD) aprE::ftsA-YFP* | SB076 transformed with YK20 |
| SB085 | *trpC2 (amyE::cam P_xyl_ minD) aprE::ftsA-YFP minJ::tet* | SB084 transformed with RD021 |
| EBS499 | *minC4-gfp, sacA::tet* | [6] |
| SB086 | *trpC2 (amyE::cam P_xyl_ minD) minC4-gfp, sacA::tet* | EBS499 transformed with SB076 |
| MB005 | *trpC2 (amyE::cam P_xyl_ gfp-minD)* | [1] |
| SB052 | *trpC2 (amyE::cam P_xyl_ gfp-minD) yvjD::tet* | MB005 transformed with RD021 |
| 1801 | *trpC2 chr:: pJSIZDpble (Pspac-ftsZ ble)* | [7] |
| SB088 | *trpC2 chr:: pJSIZDpble (Pspac-ftsZ ble) pbpB::pSG5061 (cat P_xyl_-gfp-pbpB^1−825^)* | 1801 transformed with 3122 |
| SB092 | *trpC2 chr:: pJSIZDpble (Pspac-ftsZ ble) pbpB::pSG5061 (cat P_xyl_-gfp-pbpB^1−825^) minD::erm* | SB088 transformed with SG1901 |
| SB090 | *trpC2 chr:: pJSIZDpble (Pspac-ftsZ ble) pbpB::pSG5061 (cat P_xyl_-gfp-pbpB^1−825^) yvjD::tet* | SB088 transformed with RD021 |

Table S1 references:

1. Bramkamp M, Emmins R, Weston L, Donovan C, Daniel RA, et al. (2008) A novel component of the division-site selection system of *Bacillus subtilis* and a new mode of action for the division inhibitor MinCD. Mol Microbiol 70: 1556-1569.

2. Kawai Y, Ogasawara N (2006) *Bacillus subtilis* EzrA and FtsL synergistically regulate FtsZ ring dynamics during cell division. Microbiology 152: 1129-1141.

3. Marston AL, Thomaides HB, Edwards DH, Sharpe ME, Errington J (1998) Polar localization of the MinD protein of *Bacillus subtilis* and its role in selection of the mid-cell division site. Genes Dev 12: 3419-3430.

4. Scheffers DJ, Jones LJ, Errington J (2004) Several distinct localization patterns for penicillin-binding proteins in *Bacillus subtilis*. Mol Microbiol 51: 749-764.

5. Sievers J, Errington J (2000) The *Bacillus subtilis* cell division protein FtsL localizes to sites of septation and interacts with DivIC. Mol Microbiol 36: 846-855.

6. Gregory JA, Becker EC, Pogliano K (2008) *Bacillus subtilis* MinC destabilizes FtsZ-rings at new cell poles and contributes to the timing of cell division. Genes Dev 22: 3475-3488.

7. Beall B, Lutkenhaus J (1991) FtsZ in *Bacillus subtilis* is required for vegetative septation and for asymmetric septation during sporulation. Genes Dev 5: 447-455.
