# Supplementary material for: The MinCDJ System in Bacillus subtilis Prevents Minicell Formation by Promoting Divisome Disassembly
Source: PLoS One. 2010 Mar 24;5(3):e9850. doi: 10.1371/journal.pone.0009850 (PMC2844427; doi:10.1371/journal.pone.0009850)
Supplement: Table S2 — Plasmids. (0.04 MB DOC) [file pone.0009850.s007.doc]

**Table S2: Plasmids**

| pSG1154 | *bla amyE3' spc P_xyl_ gfpmut1 amyE5'* | [1] |
| --- | --- | --- |
| pSG1186 | *bla cat cfp lacZ* | [2] |
| pSB001 | *bla cat yvjD-cfp lacZ* | YvjD_pSG1186 |
| pSB010 | *bla amyE3' spc P_xyl_ yvjD Δ243gfpmut1 amyE5'* | TM1 pSG1154 |
| pSB011 | *bla amyE3' spc P_xyl_ yvjD Δ200gfpmut1 amyE5'* | TM2 pSG1154 |
| pSB012 | *bla amyE3' spc P_xyl_ yvjD Δ130gfpmut1 amyE5'* | TM3 pSG1154 |
| pSB013 | *bla amyE3' spc P_xyl_ yvjD Δ97gfpmut1 amyE5'* | TM4 pSG1154 |
| pSB014 | *bla amyE3' spc P_xyl_ yvjD Δ57 gfpmut1 amyE5'* | TM5 pSG1154 |
| pSB016 | *bla amyE3' spc P_xyl_ yvjD Δ278 gfpmut1 amyE5'* | PDZ pSG1154 |
| pSB024 | *bla amyE3' cat P_xyl_ minC amyE5'* | MinC into pJPR1 |
| pSB025 | *bla amyE3' cat P_xyl_ minD amyE5'* | MinD into pJPR1 |

Table S2 references:

1. Lewis PJ, Marston AL (1999) GFP vectors for controlled expression and dual labelling of protein fusions in *Bacillus subtilis*. Gene 227: 101-110.

2. Feucht A, Lewis PJ (2001) Improved plasmid vectors for the production of multiple fluorescent protein fusions in *Bacillus subtilis*. Gene 264: 289-297.
