# Supplementary material for: The MinCDJ System in Bacillus subtilis Prevents Minicell Formation by Promoting Divisome Disassembly
Source: PLoS One. 2010 Mar 24;5(3):e9850. doi: 10.1371/journal.pone.0009850 (PMC2844427; doi:10.1371/journal.pone.0009850)
Supplement: Table S3 — Oligonucleotides. (0.03 MB DOC) [file pone.0009850.s008.doc]

**Table S3: Oligonucleotides**

| Oligo name | Sequence 5’🡪3’ | Restriction site |
| --- | --- | --- |
| YvjD_pSG1186_F | **CAT**CTCGAGGTGTCTTCAATG | XhoI |
| YvjD_pSG1186_R | **CAT**GAATTCTGATCCCGGCGAC | EcoRI |
| deltaTM_pSG1154_R | **GCG**GAATTCTGATCCCGAAGCGACTGCTTC | EcoRI |
| 1TM_pSG1154_F | **GCG**CTCGAGATCACAGCGAAACGCGTTTGT | XhoI |
| 2TM_pSG1154_F | **GCG**CTCGAGTTAGAATCGCACCTTAGCTGG | XhoI |
| 3TM_pSG1154_F | **GCG**CTCGAGAGATTTCCGCAAGGATTTGCA | XhoI |
| 4TM_pSG1154_F | **GCG**CTCGAGAACTCTTCGCGCAAACTGGA | XhoI |
| 5TM_pSG1154_F | **GCG**CTCGAGAACATATACAAAGGGACTGA | XhoI |
| PDZ_pSG1154_F | **GCG**CTCGAGTTAGGGCGTATTTTTCTGTCC | XhoI |
| MinC_pJPR1_F | **CAT**TCTAGAGTTGAGGTGAATATTGTG | XbaI |
| MinC_pJPR1_R | **ATG**CGGCCGTCACATTCCTCCCTCAAGCC | EagI |
| MinD_pJPR1_F | **CAT**TCTAGAggaggaatgtgaattgggtgag | XbaI |
| MinD_pJPR1_R | **ATG**CGGCCGTTAAGATCTTACTCCGAAAAATGACTT | EagI |
